# Effect of Different Surface Treatment Methods on the Shear Bond Strength of Resin Composite/Zirconia for Intra-oral Repair of Zirconia Restorations

**DOI:** 10.1055/s-0042-1756475

**Published:** 2022-10-11

**Authors:** Kamyar Fathpour, Mahsa Nili Ahmadabadi, Ramin Atash, Amir Hossein Fathi

**Affiliations:** 1 Department of Esthetic and Restorative Dentistry, Dental Material Research Center, School of Dentistry, Isfahan University of Medical Sciences, Isfahan, Iran; 2 Department of Esthetic and Restorative Dentistry, School of Dentistry, Isfahan University of Medical Sciences, Isfahan, Iran; 3 Department of Prosthodontics, School of Dentistry, Faculty of Medicine, Université Libre de Bruxelles, Brussels, Belgium; 4 Dental Materials Research Center, Dental Prosthodontics Department, School of Dentistry, Isfahan University of Medical Sciences, Isfahan, Iran

**Keywords:** zirconium, Clearfil SE bond, shear bond strength

## Abstract

**Objective** A durable resin/zirconia bond is essential for successful intra-oral repair of zirconia restorations. The purpose of this study was to evaluate the influence of two mechanical treatments followed by seven chemical treatments on the shear bond strength (SBS) of composite resin to zirconia.

**Materials and Methods** In this *in vitro* study, 280 zirconia blocks (Y-TZP) were either air-abraded or bur roughened and divided into seven experimental groups ( *n* = 20) in terms of primer/resin application: 1) ZPP, Z-Prime Plus; 2) ZPP + GP, Z-Prime Plus followed by G-premio bond; 3) ZPP + ALB, Z-Prime Plus followed by All Bond Universal; 4) ZPP + CLRF, Z-Prime Plus followed by Clearfil SE Bond; 5)GP, G-Premio Bond 6) ALB, All Bond Universal; and 7) CLRF, Clearfil SE Bond. After composite bonding and storage in distilled water (24 hours), half of each group specimen ( *n* = 10) were thermocycled. All specimens were subjected to shear force. Statistical analysis was performed using Kruskal–Wallis and Mann–Whitney test (α = 0.05).

**Results** Significant reduction in SBS was observed in all groups after thermocycling( *p* < 0.05), except for the air-abraded ZPP + CLRF ( *p* = 0.143). After aging, air-abraded CLRF exhibited the highest SBS (13.55 ± 7.8 MPa) and bur roughened ZPP showed the lowest SBS (1.16 ± 1.23 MPa). In the aged specimens, there was a significant difference between air abrasion and bur roughening in all groups ( *p* < 0.05).

**Conclusion** Air-abrasion followed by application of adhesive (with/without prior primer application) is the most efficient technique for repair of veneered zirconia restorations with resin composite.

## Introduction

In response to the increased demand for esthetic materials in modern dentistry, new framework materials have been introduced with the goal of replacing metal-ceramic restorations with more esthetically pleasing full ceramic structures. 1

Zirconia (Ytterium stabilized tetragonal zirconia or Y-TZP) is a ceramic that has received increased attention during the last decades due to its favorable mechanical properties. It is a high-strength and flaw-tolerant 2 3 biomaterial widely used in the fabrication of endodontic posts, implants and framework for crowns, and other fixed prostheses. 4

Despite the latest advances and attempts in producing more translucent zirconia restorations with monolithic systems, it is still relatively opaque. Thus, zirconia is prepared as a core material and veneered by feldspathic ceramic to achieve superior esthetics. 5

The interface between the zirconia core and the veneering porcelain is one of the weakest points in the ceramic restoration 6 ; therefore delamination and porcelain chipping with or without core exposure are one of the most frequent clinical problems encountered with zirconia-based restorations (0–44%). 7 This type of fracture can occur due to trauma, laboratory defects, and inadequate occlusal adjustment. Based on the involved area and the needed time and cost, the treatment approach varies from polishing and simple composite repair to total restoration replacement. 8 9 Fabrication of a new restoration imposes extra time and cost and additional trauma to the remaining tooth structure. 7 The advantages of direct composite repair include being less invasive and easy to perform, spending less time and cost, and also an immediate return of function and esthetics to the patient, prolonging restoration's life 10 and postponing its replacement. However, the success of such an approach largely depends on establishing a durable bond between the ceramic surface and the composite restoration. 11

Different intraoral repair techniques have been proposed for zirconia surface treatment to improve resin bonding. 7 These techniques are based on mechanical methods (e.g., grinding with bur and air particle abrasion), chemical methods (10-methacryloyloxydecyl dihydrogen phosphate [MDP] and other monomers such as 3-methacryloxypropyletrimethoxysilan [3-MPTS] 12 ) or a combined approach (e.g., tribochemical silica coating with silane primer [TBS]). 13 Several other surface treatments, such as laser irradiation and hot chemical etching, have also been explored to increase resin-to-zirconia bond strength. 14

Although comparative studies exist, showing the advantages of various types of surface conditioning methods on different ceramics, 15 these are limited in number to arrive at a consensus regarding the best surface conditioning method.

The objective of the present study was to evaluate the shear bond strength of resin-composite to Y-TZP using two different mechanical treatments followed by seven different chemical treatment protocols using zirconia primer and different bonding systems.

The null hypothesis tested was that pre-conditioning with a zirconia primer has no significant effect on the improvement of bond strength and durability when universal and sixth-generation adhesives are used for bonding to zirconia.

## Materials and Methods

In this *in vitro* experimental study, 280 partially sintered zirconia (Y-TZP) samples (IPS e.max zirCAD for inLab MO1B65/3 Stk., Ivoclar, Vivadent) with the dimensions of 12 × 12 × 5mm were sectioned with a low-concentration diamond blade (Mecatome T201 A, Presi, Grenoble, France). According to the manufacturer's instructions, the specimens were sintered at 1550°C for 10 hours in a high-temperature sintering furnace (HT-2; MIHM-VOGT GmbH, Stutensee, Germany). The final dimensions of the specimens were 9.6 × 9.6 × 4 mm following the 20% volumetric shrinkage associated with sintering. Using cylindrical metallic molds (7 mm height and 35 mm internal diameter), the fully sintered specimens were embedded in autopolymerizing acrylic resin (UNIFAST TRAD, GC, America) such that only one square surface of the specimens was left exposed for composite bonding. The exposed surface of each specimen was ground finished using the sequential application of 600 and 1200 grit silicon carbide abrasive papers (Struers RotoPol 11, Struers A/S, Rodovre, Denmark) and then placed in an ultrasonic bath of distilled water for 1 minute and steam-cleaned for 10 seconds.

Half of the specimens were submitted to air particle abrasion with 50-µm alumina particles (Rocatec, 3M) using an extra-oral air abrasion device (Dento-Prep; Ronvig, Daugaard, Denmark) at a pressure of 2.8 bars from a distance of 10 mm for 10 seconds at a 90-degree angle. The other half was roughened with a long fissure diamond bur (reference no. 0803–1, Robot Points, Shofu Inc., Kyoto, Japan, batch no. 040105) with 10 back and forth strokes with the side of the bur under water spray. The bur was replaced after five specimen preparations. After bur roughening, the specimens were rinsed with high-pressure water for 30 seconds to remove the surface debris.

Specimens receiving the same mechanical treatment were randomly divided into subgroups of 20 (7 subgroups in each of the two groups of different mechanical treatments). Thus, 14 groups were created based on different types of mechanical and chemical treatments.

Details of different chemical treatments are as follows:

1) ZPP, Z-Prime Plus; 2) ZPP + GP, Z-Prime Plus with G-premio bond; 3) ZPP + ALB, Z-Prime Plus with All Bond Universal; 4) ZPP + CLRF, Z-Prime Plus with Clearfil SE Bond (bonding resin component); 5) GP, G-Premio Bond 6) ALB, All Bond Universal; and 7) CLRF, Clearfil SE Bond (bonding resin component).

The experimental groups and the details of treatment procedures are presented in Table 1 . The materials used in this study are presented in Table 2 .

**Table 1:** Study groups based on mechanical and chemical treatment

Group Number	Mechanical treatment	Chemical treatment	Storing condition	Abbreviation
1	Air abrasion (A)	Z-Prime Plus	Thermocycled (T)	TA/ZPP
			Not thermocycled (NT)	NTA/ZPP
2		Z-Prime Plus+ G-Premio BOND	Thermocycled (T)	TA/ZPP + GP
			Not thermocycled (NT)	NTA/ZPP + GP
3		Z-Prime Plus + All Bond Universal	Thermocycled (T)	TA/ZPP + ALB
			Not thermocycled (NT)	NTA/ZPP + ALB
4		Z-Prime Plus+ Clearfil SE Bond	Thermocycled (T)	TA/ZPP + CLRF
			Not thermocycled (NT)	NTA/ZPP + CLRF
5		G-Premio BOND	Thermocycled (T)	TA/GP
			Not thermocycled (NT)	NTA/GP
6		Clearfil SE Bond	Thermocycled (T)	TA/CLRF
			Not thermocycled (NT)	NTA/CLRF
7		All Bond Universal	Thermocycled (T)	TA/ALB
			Not thermocycled (NT)	NTA/ALB
8	Bur Roughening (B)	Z-Prime Plus	Thermocycled (T)	TB/ZPP
			Not thermocycled (NT)	NTB/ZPP
9		Z-Prime Plus +G-Premio BOND	Thermocycled (T)	TB/ZPP + GP
			Not thermocycled (NT)	NTB/ZPP + GP
10		Z-Prime Plus + All Bond Universal	Thermocycled (T)	TB/ZPP + ALB
			Not thermocycled (NT)	NTB/ZPP + ALB
11		Z-Prime Plus + Clearfil SE Bond	Thermocycled (T)	TB/ZPP + CLRF
			Not thermocycled (NT)	NTB/ZPP + CLRF
12		G-Premio Bond	Thermocycled (T)	TB/GP
			Not thermocycled (NT)	NTB/GP
13		Clearfil SE Bond	Thermocycled (T)	TB/CLRF
			Not thermocycled (NT)	NTB/CLRF
14		All Bond Universal	Thermocycled (T)	TB/ALB
			Not thermocycled (NT)	NTB/ALB

Abbreviations: A, air abrasion; ALB, All Bond Universal; B, bur roughening; CLRF, Clearfil SE Bond; GP, G-Premio BOND; NT, not thermocycled; T, thermocycled; ZPP, Z-Prime Plus.

**Table 2:** Materials used in the study

Material/Trade name	Manufacturer	Abbreviation	Main compositions	Application method
IPS e.max ZirCADa	Ivoclar Vivadent		ZrO2 (87.0-95.0wt%), Y2O3 (4.0-6.0wt%), HfO2 (1.0-5.0wt%), Al2O3 (0.1-1.0wt%), Other oxides (,0.2wt%)	
Z-Prime Plus	Bisco Inc	ZPP	Ethanol 75-85%, BisGMA 5-10%, HEMA 5-10%, organophosphate monomer (MDP) 1-5%, carboxylic acid resin monomer	Two coats were applied and each layer was gently air-dried for 5 seconds and the second layer was light-cured for 10 seconds.
Clearfil SE Bond (adhesive part)	Kuraray Medical Inc, Okayama, Japan	CLRF	10-MDP, HEMA, Bis-GMA, hydrophobic dimethacrylate, photoinitiator, silanated colloidal silica	One layer of the bond was applied and evenly distributed with mild airflow and light-cured for 10 seconds
G-Premio BOND	GC, Tokyo, Japan	GP	10-MDP, Acetone, dimethacrylate component, photoinitiator, butylated hydroxytoluene, water, silica	One layer was applied, after 10 seconds it was dried for 5 seconds with max air pressure, and then it was light-cured for 10 seconds
All Bond Universal	Bisco	ALB	10-MDP, Bis-GMA, HEMA, ethanol, water,initiators.	One layer of adhesive was applied and the solvent was evaporated with air-drying followed by light curing for 10 seconds

After mechanical treatment, chemical treatment was performed to a limited area of 2 mm in diameter of each specimen, standardized with perforated adhesive tape (Adhesive Vinyl, SRA3; Xerox Labels, Antalis, UK). In subgroups with Z-Prime Plus as the only chemical treatment, this primer was applied in two coats, air-dried, and then, it was light-cured. In the subgroups where an adhesive was applied after Z-Prime Plus, the primer was not light-cured and photo-polymerization was done after adhesive application. A plastic cylinder (1.5 mm diameter, 2 mm height) was placed in the center of the prepared area of each specimen and resin composite (Charisma Diamond, Kulzer, Germany), shade A2, was placed, and condensed into the plastic mold and filled up in two layers. Each layer was light-polymerized for 40 seconds. All curing steps were performed using a light-curing unit (VALO, Ultradent Products Inc, South Jordan, UT, USA) operating at 1000 mW/cm ^2^ (standard power) light intensity, as measured by a radiometer. The bonded specimens were then stored in distilled water at 37°C for 24 hours. Then, half of the samples in each group were subjected to thermal cycling for 10,000 cycles between 5°C and 55°C with 25 seconds of dwell time. Finally, the shear bond test was performed using a universal testing machine (Zwick ROELL Z2.5 MA 18–1-3.7uim, Germany). The bonding surface was parallel to the loading device and a knife-edge indenter exerted shear load at the composite-zirconia interface as close as possible to the interface with a crosshead speed of 1 mm/min. Load at failure was recorded in MPa and the mean shear bond strength of each group was calculated. Data were analyzed using Kruskal–Wallis and Bonferroni's post hoc test. A *p* -value< 0.05 was considered significant.

Failure type analysis:

The mode of failure was evaluated under a stereomicroscope (Zeiss, Jena, Germany) at 20× magnification by two observers blinded to the experimental conditions.

The mode of failure was classified as follows:

1. Adhesive failure (A): failure at the interface between the adhesive and zirconia surface
2. Cohesive failure (C): failure in the bulk of composite
3. Mixed failure (M): a combination of adhesive failure and cohesive failure in composite bulk.

## Results

The mean shear bond strength (SBS) values in MPa and standard deviations of the study groups are presented in Table 3 . Kruskal–Wallis test revealed significant differences between different chemical treatment methods regardless of aging and type of mechanical treatment ( *p* < 0.001).

**Table 3:** Mean shear bond strength values and standard deviation (SD) of experimental groups

Mechanical treatment	Chemical treatment	Mean shear bond strength (MPa) ± SD		*P* -value
		No Thermocycling	Thermocycling	
Bur roughening (B)	ZPP	**(** 13.65 **) ± ** 24.56	**(** 1.05 **) ± ** 1.16	0.009
	ZPP + GP	**(** 7.40 **) ± ** 20.57	**(** 1.07 **) ± ** 1.38	<0.001
	ZPP + ALB	**(** 6.88 **) ± ** 30.05	**(** 3.47 **) ± ** 4.62	<0.001
	ZPP + CLRF	**(** 5.47 **) ± ** 34.90	**(** 3.42 **) ± ** 5.83	<0.001
	GP	**(** 4.86 **) ± ** 19.75	**(** 1.60 **) ± ** 2.35	<0.001
	ALB	**(** 5.32 **) ± ** 16.63	**(** 0.92 **) ± ** 2.07	<0.001
	CLRF	**(** 10.04 **) ± ** 24.76	**(** 3.20 **) ± ** 5.05	<0.001
Air abrasion (A)	ZPP	**(** 9.80 **) ± ** 24.46	**(** 2.09 **) ± ** 3.83	<0.001
	ZPP + GP	**(** 4.94 **) ± ** 18.82	**(** 2.66 **) ± ** 7.49	<0.001
	ZPP + ALB	**(** 5.08 **) ± ** 18.66	**(** 3.60 **) ± ** 10.35	<0.001
	ZPP+ CLRF	**(** 7.85 **) ± ** 20.55	**(** 4.78 **) ± ** 11.78	* 0.143
	GP	**(** 12.10 **) ± ** 18.62	**(** 5.18 **) ± ** 7.07	<0.001
	ALB	**(** 4.99 **) ± ** 17.46	**(** 4.36 **) ± ** 10.85	<0.001
	CLRF	**(** 5.47 **) ± ** 30.34	**(** 6.80 **) ± ** 13.55	<0.001

*Not Significant.

**Fig. 1:**
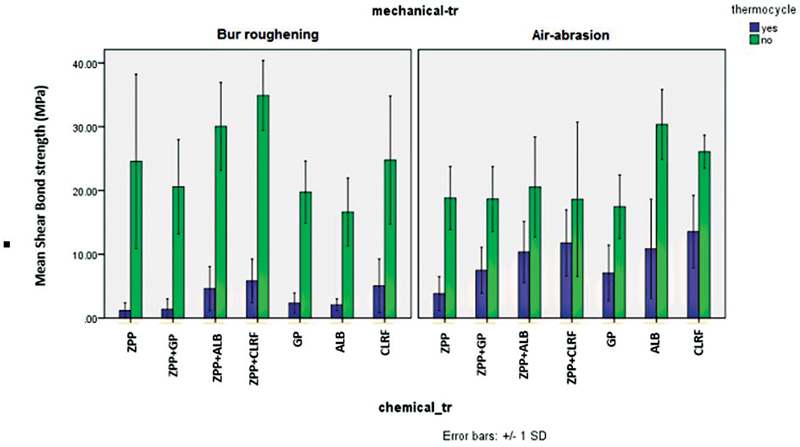
Mean values for shear bond strength in all experimental groups before and after thermocycling. ZPP: Z-Prime Plus; GP: G-Premio Bond, ALB: All Bond Universal; CLRF: Clearfil SE Bond; tr: treatment.

The results of Bonferroni post hoc tests revealed a significant reduction in shear bond strength (SBS) in all groups after thermocycling except for air-abraded ZPP + CLRF ( *p* = 0.143) ( Table 3 and Fig. 1 ).

After being thermocycled, the A/CLRF group exhibited the highest SBS (13.55 ± 6.8 MPa) and B/ZPP group showed the lowest SBS (1.16 ± 1.05 MPa) among the experimental groups ( Table 3 ). There was a significant difference between air abrasion and bur roughening methods in all groups after thermocycling ( *p* < 0.05) ( Table 4 ).

**Table 4:** Comparison of shear bond strength between different groups considering type of mechanical treatment

Chemical treatment	Not thermocycled (NT)	Thermocycled (T)
ZPP	0.043*	0.01*
ZPP + GP	0.436	<0.001*
ZPP + ALB	0.015*	0.004*
ZPP + CLRF	0.002*	0.007*
GP	0.529	0.001*
ALB	<0.001*	0.001*
CLRF	0.971	0.004*

*Significant difference in SBS.

**Fig. 2:**
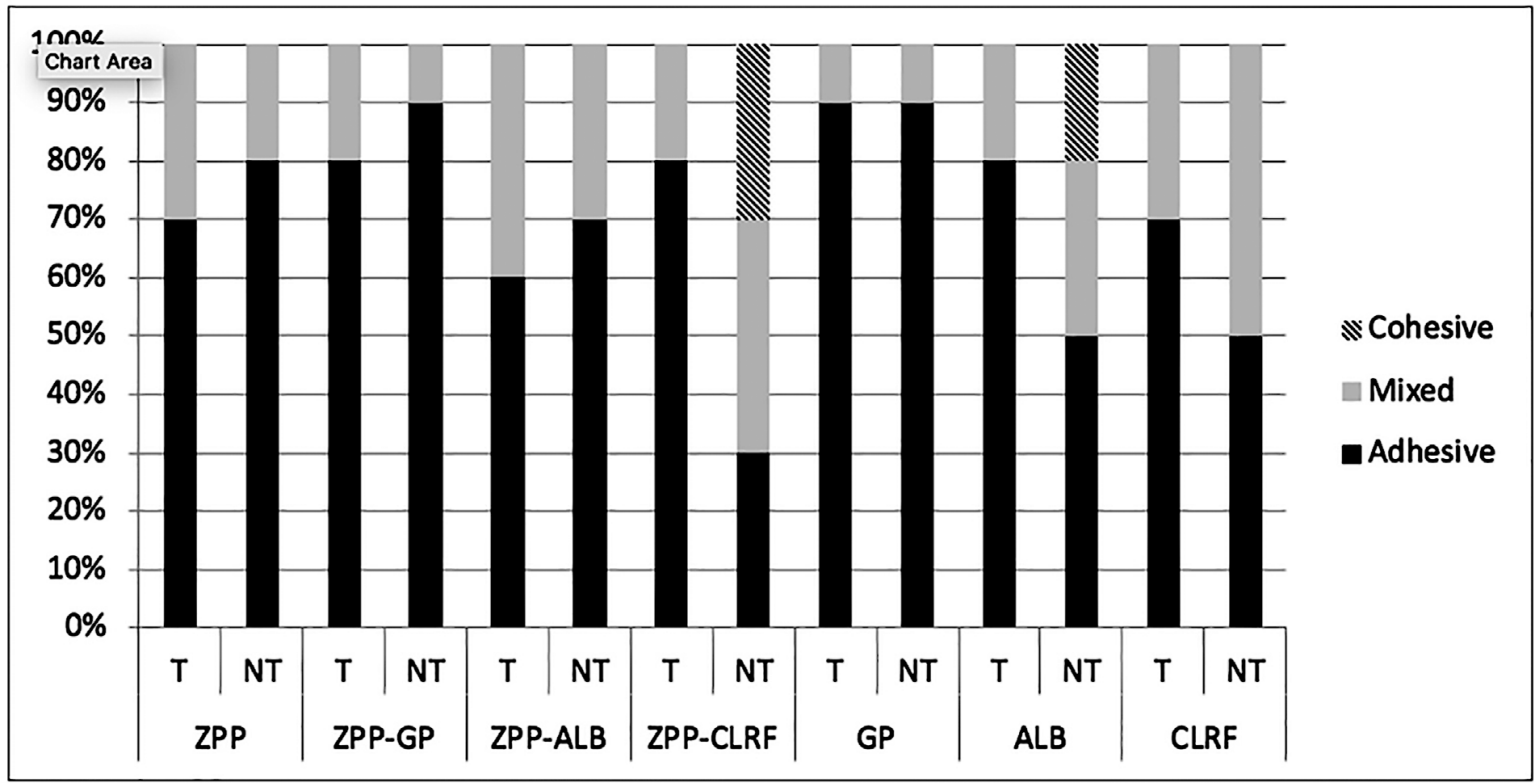
Mode of failure in air abraded groups before and after thermocycling. T: thermocycled: NT: not thermocycled; ZPP: Z-Prime Plus; GP: G-Premio Bond, ALB: All Bond Universal; CLRF: Clearfil SE Bond.

**Fig. 3:**
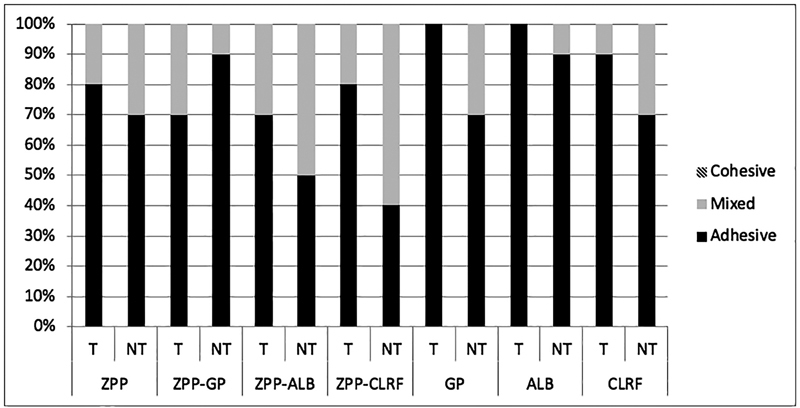
Mode of failure in bur-roughening groups before and after thermocycling. T: Thermocycled: NT: Not Thermocycled. ZPP: Z-Prime Plus; GP: G-Premio Bond, ALB: All Bond Universal; CLRF: Clearfil SE Bond.

**Fig. 4:**
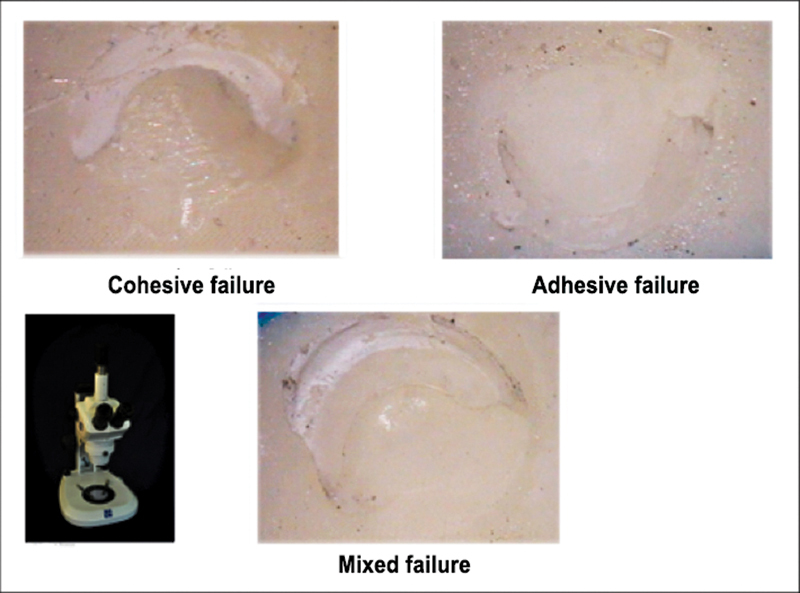
Different modes of failure.

The mode of failure in different groups is presented in Figs. 2 and 3 based on the mechanical treatment method. Failure mode was mostly adhesive (60–100%). Cohesive failure was only observed in NTA/ALB and NTA/ ZPP + CLRF groups (there was no cohesive failure for bur-roughening subgroups). In both groups of TB/GP and TB/ALB, 100% failures were adhesive ( Fig. 3 ). Examples of images of different modes of failures are given in Fig. 4 .

## Discussion

This study evaluated different combinations of mechanical (air abrasion/bur roughening) and chemical treatments using zirconia primer and different bonding systems on the shear bond strength of composite to zirconia before and after thermocycling. According to the results of this study, thermocycling significantly reduced SBS in all groups except for A/ZPP + CLRFL ( Table 3 ).

Based on our findings, air abrasion resulted in a more durable bond than bur roughening after thermocycling ( Fig. 1 ). In terms of different chemical treatments, the A/ZPP + CLRFL group was the only group in which SBS was not significantly reduced after thermocycling ( *p* = 0.143) ( Table 3 ). After thermocycling, A/CLRFL showed the highest SBS and B/ZPP the lowest SBS ( Table 3 ).

In a study by Cristoforides, 16 air abrasion was not effective in improving bond strength between resin composite and Y-TZP zirconia; however, there are several other short-term reports, 17 18 confirming that air abrasion improved the bond strength of composite resin to zirconia.

Considering the controversial effect of air abrasion on the zirconia surface and taking into account that the use of bur for zirconia roughening is easier and more convenient compared with the unfavorable condition accompanied by air abrasion, these two mechanical treatment methods were evaluated in this study.

In a study by Suliman et al evaluating the effect of various surface treatments on porcelain repair, the findings indicated that bur-roughening was the most effective mechanical treatment; however it was not significantly better than air-abrasion. 19

In a study evaluating the durability of the bond between direct composite resin and zirconia, Attia 20 reported that air-abrasion demonstrated a higher bond strength than bur roughening, which is consistent with our findings. On the basis of our results, as samples were subjected to thermocycling, a significant difference was observed between two mechanical treatments in all the seven groups and air-abrasion demonstrated significantly higher bond strength than bur-roughening ( Fig. 1 ), indicating a more durable bond.

In a study by Libecki et al, 1 treatment with both air-abrasion and bur-roughening resulted in high initial bond strength; however, after aging, the air-abraded group showed less reduction in bond strength and hence a more durable bond while bur-roughening resulted in significant reduction but still acceptable bond.

In line with these studies, our findings showed that compared with bur-roughening, air-abrasion results in a more durable bond. In the bur-roughened samples, the bond strength in all specimens reduced below the acceptable clinical level after thermocycling, 21 while among the air-abraded samples there were four groups (ZPP + CLRF, CLRFL, ZPP + ALB, ALB) that showed bond strength in the range of acceptable clinical bond strength (10–13MPa) 21 ( Table 3 ).

As previously mentioned, in the current study after mechanical treatment, chemical treatment was performed on each specimen, assessing the ability of three adhesives: two Universal MDP-containing adhesives (All Bond Universal and G-Premio Bond) and the bonding component of one self-etch adhesive system (Clearfil SE Bond) in the presence or absence of pretreatment with Z-Prime Plus (MDP-containing zirconia primer) to establish a stable bond between the direct composite and zirconia ceramic.

Recently a wide variety of one-bottle universal adhesives have been designed, giving the clinicians a wide range of options for having a successful bond to almost all types of restorative materials. 22 Manufacturers claim that the presence of components such as 10-MDP makes it possible to have a stable bond to these surfaces without prior use of a primer. 22 Because the degree of the chemical bond provided by 10-MDP depends on the concentration of this monomer, the chemical bond strength of universal adhesives is much weaker compared with an adhesive such as Clearfil SE bond that contains higher concentrations of this monomer. 23 Furthermore, the presence of HEMA in the formulation of some universal adhesives might compromise the strength of the chemical bond achieved with 10-MDP. 24

Amaral 15 and Seabra 25 reported that the application of one-bottle universal adhesives alone provides higher bond strength to zirconia compared with using zirconia primers alone. These findings are in agreement with our results ( Table 3 ) ( Fig. 1 ).

There is not enough information on whether the application of zirconia primers before adhesives could result in a stronger and more durable bond. It has been claimed that the new generation of universal adhesives makes the bond to zirconia possible without prior application of primer. 15

On the basis of his study, Kim concluded that the application of universal adhesives could replace the application of primers alone. Based on our results, the application of universal or sixth-generation adhesive (with or without prior primer application) resulted in higher bond strengths after thermocycling compared with the groups in which only the primer was applied. The probable reason could be that in contrast to primers, adhesives contain more hydrophobic monomers that might reinforce the interface by co-polymerizing with the composite resin. 26

In our study, we concluded that although the application of Z-Prime Plus alone resulted in an acceptable initial bond strength (24.5 MPa), the bond strength was significantly reduced after thermocycling ( Table 3 ). This result suggests that to achieve a durable strong bond, Z-Prime Plus should not be used alone and it is necessary to apply an additional adhesive layer.

The findings of this study indicate that although the application of Z-Prime Plus before some adhesives (Clearfil SE Bond and All Bond Universal) significantly increased the initial bond strength, this increased bond strength diminished significantly with thermocycling and the bond strength difference was not significant whether or not primer was applied before adhesive ( Table 5 ). Therefore, it could be concluded that although the application of Z-Prime Plus before the adhesive layer could have a positive effect on initial bond strength, this effect is significantly reduced with aging ( Table 3 ).

**Table 5:** Comparison of shear bond strength between different groups considering the type of chemical treatment

Chemical treatment	Not thermocycled (NT)		Thermocycled (T)	
	Bur roughening	Air-abrasion	Bur roughening	Air-abrasion
ZPP & ZPP + GP	0.123	0.971	1	* 0.01
ZPP & ZPP + ALB	0.436	0.912	* 0.005	* 0.001
ZPP & ZPP + CLRF	* 0.043	0.853	* 0.001	* 0.001>
ZPP & GP	* 0.043	0.739	0.105	0.059
ZPP & ALB	* 0.035	* 0.001>	0.075	* 0.007
ZPP & CLRF	0.796	* 0.002	* 0.002	* 0.001
ZPP + GP & ZPP + ALB	* 0.01	0.739	* 0.035	0.105
ZPP + GP & ZPP + CLRF	* 0.001>	0.912	* 0.003	* 0.023
ZPP + GP & GP	0.684	0.739	0.247	0.529
ZPP + GP & ALB	0.247	* 0.001>	0.315	0.247
ZPP + GP & ClRF	0.315	* 0.001	* 0.007	* 0.009
ZPP + ALB & ZPP + CLRF	0.089	1	0.393	0.912
ZPP + ALB & GP	* 0.002	0.631	0.280	0.089
ZPP + ALB & ALB	* 0.001>	* 0.009	0.075	0.631
ZPP + ALB & CLRF	0.190	0.052	0.579	0.143
ZPP+ CLRF & GP	* 0.001>	0.684	* 0.019	* 0.019
ZPP+ CLRF & ALB	* 0.001>	* 0.015	* 0.004	0.579
ZPP + CLRF & CLRF	* 0.023	0.089	0.393	0.280
GP & ALB	0.190	* 0.001>	0.579	0.165
GP & CLRF	0.190	* 0.001>	0.089	* 0.015
CLRF & ALB	0.052	0.052	* 0.009	0.075

*Significant difference in SBS.

A possible explanation could be that because Z-Prime Plus is a very low viscosity fluid and contains a high concentration of ethanol, rendering it highly volatile, it is possible that the pressure caused by the composite application causes extrusion of the primer from the interface. As a result, in cases where only the primer was applied, the remaining primer is not enough to establish an adequate bond. 25 When a layer of adhesive resin is applied over the primer and then light-cured, higher bond strength is achieved because a more stable interface is formed between zirconia and composite resin.

The bonding component of the Clearfil SE Bond adhesive contains a hydrophobic resin monomer that reinforces the interfacial layer by co-polymerizing with the overlying resin composite. It also contains a 10-MDP monomer that positively affects the interfacial bond strength by creating a chemical bond to different substrates. This layer of resin monomer could reduce the hydrolytic instability caused by the presence of HEMA, which is one of the components of ZPP. 27 This may explain the higher bond strength after thermocycling in groups where ZPP was applied followed by a layer of adhesive resin ( Table 3 ).

A comparison of different groups based on the type of universal adhesive used showed that ALB mostly resulted in higher repair SBS compared with GP ( Fig. 1 ). A possible reason could be the difference in the type of solvents present in each adhesive. ALB contains ethanol and GP contains acetone. Because acetone has a lower boiling temperature (56.5°C) and higher vapor pressure (200 mm Hg) compared with ethanol, after solvent evaporation a thinner adhesive layer is left, which is more prone to polymerization inhibition by oxygen and it could negatively influence the bond strength at the interface. 28 This problem might be overcome by the application of multiple layers to result in a thicker adhesive layer.

In a study evaluating the shear bond strength of composite resin to zirconia, Shafiei concluded that the addition of a resin layer over the applied primer (Z-Prime Plus) increased bond strength significantly. Application of Z-Prime Plus followed by Clearfil SE bond (bonding component) showed the highest shear bond strength in her study. 29 All specimens were prepared by air abrasion before chemical treatment.

The findings of Shafiei's study are in agreement with our results. In our study, among all thermocycled groups, the highest shear bond strength was obtained in TA/CLRFL (13.55 MPa) group and TA/ZPP + CLRFL(11.78MPa) ( Table 3 ). Moreover, the shear bond strength in TA/ZPP + ALB (10.35MPa) and TA/ALB (10.85 MPa) remained at the minimum acceptable level (10–13MPa), while the bond strength was lower in other groups ( Table 3 ).

In a study by Mahgoli et al 30 assessing the efficacy of two zirconia primers (Z-Prime Plus and Monobond Plus) and Porcelain Bonding Resin (PBR) for the intraoral repair of zirconia restorations these three agents were applied separately and in combination. The results showed the maximum SBS for ZPP + PBR followed by MBP + PBR. They concluded that the additional layer of resin (PBR) increased the SBS significantly. The finding that higher bond strength is achieved when a resin layer is applied over the primer is in line with our results. This could be explained by the hydrophobicity of the resin monomer that reinforces the interfacial layer by co-polymerizing with the overlying resin composite.

In our study, the mode of failure was mostly adhesive (60–100%). Cohesive failure was only observed in NTA/ALB and NTA/ZPP + CLRF groups. In both groups of TB/GP and TB/ALB, 100% of the failures were adhesive ( Figs. 2 and 3 ).

In general, the reported bond strength from different studies could not be directly compared due to various study conditions such as different test methods, bonding surface area, and aging protocols. 29

There are clearly some limitations with this study. In the clinical situation when chipping occurs in the veneered zirconia crowns, the surface to be repaired comprises two different substrates; zirconia and surrounding porcelain, with the porcelain being susceptible to hydrofluoric acid etching. Probably in the clinical situation, the bond strength is higher due to the presence of etchable porcelain. Due to the presence of complex forces, tests such as cyclic loading could better simulate intra-oral conditions compared with shear bond strength tests. Furthermore, ceramic restorations are subjected to a combination of mechanical and thermal stresses, whereas, in the current study only the thermal stress test was conducted. PH fluctuation and chemical challenges caused by salivary enzymes were not considered in this study. Another limitation to consider was that zirconia samples were not subjected to aging (resemblance to the clinical situation) before performing the bonding procedures. It has been shown that this factor could significantly reduce the bond strength to zirconia. 31

## Conclusions

Within the limitations of this study, it can be concluded that

1. Thermocycling significantly reduced SBS in all studied groups (except for the air-abraded ZPP + CLRF).
2. Air-abrasion results in a more durable bond than bur-roughening.
3. Considering all the adhesives used in this study, the combined application of primer-adhesive or application of adhesive alone significantly increases the SBS of composite resin to zirconia compared with primer alone.
4. Application of primer before adhesive does not have a significant effect on the shear bond strength of composite resin to zirconia.
5. Among the different assessed chemical treatments, Clearfil SE Bond and Z-Prime Plus + Clearfil SE Bond resulted in the highest SBS of composite resin to zirconia.

## References

[1] Libecki W Elsayed A Lehmann F Kern M Efficacy of different surface treatments for tntraoral repair of veneered zirconia frameworks J Adhes Dent 2017 19 04 323 329 28849798 10.3290/j.jad.a38891

[2] Kelly J R Benetti P Ceramic materials in dentistry: historical evolution and current practice Aust Dent J 2011 56(Suppl 1):84 96 21564119 10.1111/j.1834-7819.2010.01299.x

[3] Peampring C Kengtanyakich S Surface roughness and translucency of various translucent zirconia ceramics after hydrothermal aging Eur J Dent 2022 16 04 761 767 34891182 10.1055/s-0041-1736415 PMC9683892

[4] Subaşı M G İnan Ö Evaluation of the topographical surface changes and roughness of zirconia after different surface treatments Lasers Med Sci 2012 27 04 735 742 21786139 10.1007/s10103-011-0965-3

[5] Tokar E Polat S Ozturk C Repair bond strength of composite to Er,Cr:YSGG laser irradiated zirconia and porcelain surfaces Biomed J 2019 42 03 193 199 31466713 10.1016/j.bj.2019.02.001 PMC6717752

[6] Aboushelib M N de Jager N Kleverlaan C J Feilzer A JJDM Microtensile bond strength of different components of core veneered all-ceramic restorations Dent Mater 2005 21 10 984 991 16085302 10.1016/j.dental.2005.03.013

[7] Agingu C Zhang C-y Jiang N-w Intraoral repair of chipped or fractured veneered zirconia crowns and fixed dental prosthesis: clinical guidelines based on literature review. Journal of Adhesion Science and Technology (Singap) 2018 32 15 1711 1723

[8] Hickel R Brüshaver K Ilie N Repair of restorations–criteria for decision making and clinical recommendations Dent Mater 2013 29 01 28 50 22867859 10.1016/j.dental.2012.07.006

[9] Mesquita A MM Al-Haj Husain N Molinero-Mourelle P Özcan M An intraoral repair method for chipping fracture of a multi-unit fixed zirconia reconstruction: a direct dental technique Eur J Dent 2021 15 01 174 178 33622015 10.1055/s-0040-1716311 PMC7902107

[10] Kumbuloglu O User A Toksavul S Vallittu P KJAOS Intra-oral adhesive systems for ceramic repairs: a comparison Acta Odontol Scand 2003 61 05 268 272 14763777 10.1080/00016350310005556

[11] Arami S Hasani Tabatabaei M Namdar F Safavi N Chiniforush N Shear bond strength of the repair composite resin to zirconia ceramic by different surface treatment J Lasers Med Sci 2014 5 04 171 175 25653817 PMC4281985

[12] Li R Wang C Ma S Q High bonding strength between zirconia and composite resin based on combined surface treatment for dental restorations J Appl Biomater Funct Mater 2020 18 2.280800020928655E15 10.1177/2280800020928655 33147097

[13] Xie H Li Q Zhang F Comparison of resin bonding improvements to zirconia between one-bottle universal adhesives and tribochemical silica coating, which is better? Dent Mater 2016 32 03 403 411 26754430 10.1016/j.dental.2015.12.014

[14] Casucci A Osorio E Osorio R Influence of different surface treatments on surface zirconia frameworks J Dent 2009 37 11 891 897 19616882 10.1016/j.jdent.2009.06.013

[15] Amaral M Belli R Cesar P F Valandro L F Petschelt A Lohbauer U The potential of novel primers and universal adhesives to bond to zirconia J Dent 2014 42 01 90 98 24246687 10.1016/j.jdent.2013.11.004

[16] Cristoforides P Amaral R May L G Bottino M A Valandro L F Composite resin to yttria stabilized tetragonal zirconia polycrystal bonding: comparison of repair methods Oper Dent 2012 37 03 263 271 22313269 10.2341/11-193-L

[17] Wolfart M Lehmann F Wolfart S Kern M Durability of the resin bond strength to zirconia ceramic after using different surface conditioning methods Dent Mater 2007 23 01 45 50 16427692 10.1016/j.dental.2005.11.040

[18] Oyagüe R C Monticelli F Toledano M Osorio E Ferrari M Osorio R Effect of water aging on microtensile bond strength of dual-cured resin cements to pre-treated sintered zirconium-oxide ceramics Dent Mater 2009 25 03 392 399 18952276 10.1016/j.dental.2008.09.002

[19] Suliman A-HA Swift E J Jr Perdigao J Effects of surface treatment and bonding agents on bond strength of composite resin to porcelain J Prosthet Dent 1993 70 02 118 120 8396642 10.1016/0022-3913(93)90004-8

[20] Attia A Influence of surface treatment and cyclic loading on the durability of repaired all-ceramic crowns J Appl Oral Sci 2010 18 02 194 200 20485932 10.1590/S1678-77572010000200015 PMC5349757

[21] Lüthy H Loeffel O Hammerle C HJDM Effect of thermocycling on bond strength of luting cements to zirconia ceramic Dent Mater 2006 22 02 195 200 16143382 10.1016/j.dental.2005.04.016

[22] Perdigão J Swift E J Jr Universal adhesives J Esthet Restor Dent 2015 27 06 331 334 26767920 10.1111/jerd.12185

[23] Tian F Zhou L Zhang Z Paucity of nanolayering in resin-dentin interfaces of MDP-based adhesives J Dent Res 2016 95 04 380 387 26701351 10.1177/0022034515623741 PMC4802780

[24] Yoshida Y Yoshihara K Hayakawa S HEMA inhibits interfacial nano-layering of the functional monomer MDP J Dent Res 2012 91 11 1060 1065 22968157 10.1177/0022034512460396

[25] Seabra B Arantes-Oliveira S Portugal J Influence of multimode universal adhesives and zirconia primer application techniques on zirconia repair J Prosthet Dent 2014 112 02 182 187 24445031 10.1016/j.prosdent.2013.10.008

[26] Kim J H Chae S Y Lee Y Han G J Cho B H Effects of multipurpose, universal adhesives on resin bonding to zirconia ceramic Oper Dent 2015 40 01 55 62 25084107 10.2341/13-303-L

[27] Lopes G C Spohr A M De Souza G M Different strategies to bond Bis-GMA-based resin cement to zirconia J Adhes Dent 2016 18 03 239 246 27200434 10.3290/j.jad.a36137

[28] Reis A Loguercio A D A 36-month clinical evaluation of ethanol/water and acetone-based etch-and-rinse adhesives in non-carious cervical lesions Oper Dent 2009 34 04 384 391 19678442 10.2341/08-117

[29] Shafiei F Fattah Z Kiomarsi N Dashti M H Influence of primers and additional resin layer on zirconia repair bond strength J Prosthodont 2019 28 07 826 832 30582263 10.1111/jopr.13011

[30] Mahgoli H Arshad M Rasouli K Sobati A A Shamshiri A R Repair bond strength of composite to zirconia ceramic using two types of zirconia primers Front Dent 2019 16 05 342 350 32123874 10.18502/fid.v16i5.2279 PMC7040558

[31] Perdigão J Fernandes S D Pinto A M Oliveira F A Effect of artificial aging and surface treatment on bond strengths to dental zirconia Oper Dent 2013 38 02 168 176 22788723 10.2341/11-489-L

